# Polyculture and Monoculture Affect the Fitness, Behavior and Detoxification Metabolism of *Bemisia tabaci* (Hemiptera: Aleyrodidae)

**DOI:** 10.3389/fphys.2018.01392

**Published:** 2018-10-04

**Authors:** Ning Di, Kai Zhang, Fan Zhang, Su Wang, Tong-Xian Liu

**Affiliations:** ^1^State Key Laboratory of Crop Stress Biology for Arid Areas, and Key Laboratory of Northwest Loess Plateau Crop Pest Management of Ministry of Agriculture, Northwest A&F University, Xianyang, China; ^2^Institute of Plant and Environment Protection, Beijing Academy of Agriculture and Forestry Sciences, Beijing, China; ^3^Development Center for Science and Technology, Ministry of Agriculture and Rural Affairs of the People’s Republic of China, Beijing, China

**Keywords:** polyculture, monoculture, host plant diversity, polyphagous, *Bemisa tabaci*

## Abstract

Herbivores respond differently to the level of plant diversity encountered. *Bemisia tabaci* Gennadius (Hemiptera: Aleyrodidae) are highly polyphagous herbivores which cause considerable damage to various crops. Herein, we reared this species both in polyculture and monoculture, including preferred and less preferred host plants such as Chinese cabbage (*Brassica rapa* L.), tomato (*Solanum lycopersicum* L.), kidney bean (*Phaseolus vulgaris* L.) and summer squash (*Cucurbita pepo* L.). Trends in survival and oviposition were recorded, and impact of plants on growth and development of *B. tabaci* were studied, particularly in terms of detoxification and digestive enzymatic activity in the insects. We found that the survival rate was the highest in Chinese cabbage monoculture treatment. Further, the egg numbers on individual species in the polyculture generally reflected numbers on the same plant species in monoculture. However, more eggs were observed in each of the four plant species tested in the context of polyculture. The activity of superoxide dismutases (SOD) and alkaline phosphatase (AKP) in *B. tabaci* fed in a choice situation were significantly lower than those fed with tomato monoculture, indicating a dilution of toxicity with a multi-plant diet compared with less preferred host plant diet. Also, the survival rate of *B. tabaci* in monoculture was negatively correlated with SOD amount of whitefly. In the plants attacked by whiteflies, the activity of polyphenol oxidase (PPO) and catalase (CAT) in Chinese cabbage was lower in polyculture than in the monoculture. These results implied that multi-plant treatments contained fewer secondary metabolite substances and might be less toxic to polyphagous herbivores. As such, the work herein contributes knowledge relevant for more effective control and management of *B. tabaci*.

## Introduction

Numerous studies have shown a mixed diet benefits various wildlife, including herbivores (Kitting, 1980; Lobel and Ogden, 1981; Belovsky, 1984; Dearing and Schall, 1992; Guglielmo et al., 1996). Diet breadth can be constrained by the size, mobility, Information seeking capability and neural structure of an insect, as well as habitat (Cates, 1980; Zhang et al., 2014). Further, a complex sensory environment can complicate the process of making a feeding selection, or cause distractions from feeding.

Depending on their host range, insects may be classified as monophagous, oligophagous or polyphagous (Zhang et al., 2014). Studies in plant-herbivore interactions suggest that monophagous insects undergo behavioral and physiological adaptations to a specific nutritional resource; polyphagous strategy holds the advantage of reduced time in searching for food, greater choice and higher likelihood of enemy-free spaces, nutritional complementation and toxin dilution (Freeland and Janzen, 1974; Guglielmo et al., 1996), while oligophagy carries the risk of starvation if the preferred host plant is not available. *Bemisia tabaci* (Gennadius) (Hemiptera: Aleyrodidae), which is widely distributed worldwide in tropical and subtropical climate, and also occupying some temperate habitats, is extremely polyphagous, as it feeds on over 900 plant species (Oliveira et al., 2001). It threatens the growth of vegetables and crops, and causes severe economic losses in agriculture (Zang and Liu, 2010; De Barro et al., 2011; Chu et al., 2012; Liu et al., 2012; Jiao et al., 2013). However, with a variety of both preferred and less preferred host plants of the polyphagous insects, the food source selection behavior and suitability of those insects are significantly affected by the proportion and distribution of preferred or non-preferred hosts (Cates, 1980). Thus, it is essential to understand responses of *B. tabaci* to host polyculture, as this could provide a more solid foundation for environmentally friendly pest management (Zhang et al., 2014).

It has been documented that factors in plant selection by herbivores may include host suitability (Liu and Stansly, 1995; Zhu et al., 2005), host distribution (Ballabeni et al., 2003), interspecific competition and predation (Mayhew, 1997; Gripenberg et al., 2010; Rodriguez-Saona et al., 2010), as well as the previous feeding experience of the herbivores (Papaj and Prokopy, 1989). Two main theories have been proposed to account for the evolutionary and ecological factors influencing host plant choice: the optimal oviposition theory and the optimal foraging theory (Scheirs and De Bruyn, 2002; Scheirs et al., 2004). Researchers have focused on the performance or the biochemical reaction of herbivorous insects to a specific plant or multi-plant diet, aiming to understand the evolutionary relationships between them (Mayhew, 2001; Scheirs and De Bruyn, 2002; Jiao et al., 2013; Zhang et al., 2014). One example is that growth rate of *Schistocerca americana* L. given a mixture of different plants is higher than monoculture (Bernays and Minkenberg, 1997). Also, polyculture can be beneficial for *Malacosoma castrensis* L. and *Nezara viridula* L. (Kester and Smith, 1984). However, a study on *Lymantria dispar* L. larvae has shown that the fitness on multi-plant species equals single-plant species (Stoyenoff et al., 1994). In addition, different host plants benefit different instars, thus the fitness of herbivores may increase by host switch during the growth period (Barbosa et al., 1986).

There has been some debate on these observations. One suggestion is that polyphagous individuals have interrupted decision making when presented with choices in host plant. The increased time required for foraging in this situation leads to the decreased feeding time, which thus impacts colony growth (Dall and Cuthill, 1997). More rapid population expansion can be fostered by enhanced performance of *B. tabaci* due to the longer settlement in monoculture (Bernays, 1999). However, an alternative explanation suggests that even within plant mixtures, it is the predominant effect of foraging on the most beneficial host plants that result in high fecundity of *B. tabaci* (Bird and Krüger, 2006). It might be assumed that the females prefer ovipositing on the species which could result in better outcomes for their offspring. However, in some studies this was not observed, an unexpected finding which requires further investigation (Thompson, 1988; Mayhew, 1997; Berdegué et al., 1998; Gripenberg et al., 2010; Jiao et al., 2012).

The co-evolution of insect herbivores and their host plants involves the development of resistance strategies in both parties. There are a variety of enzymes in whiteflies which function both in insect nutrition and development as well as adaptation to host plants. The activity of digestive enzymes in *B. tabaci* reflects their effectiveness for the particular host plant encountered. Secondary metabolites in plants are capable of manipulating the level of secretion of digestive enzymes in insects, either to increase or reduce (Howe and Jander, 2008; Zhang et al., 2008). Thus, it is essential to test the digestive and detoxification substances in *B. tabaci* and the host plants, which will provide information on the response mechanisms of polyphagous insects in the presence of multiple hosts. For instance, sucrase and amylase, secreted by digestive glands, both digest nutrients (Jones et al., 1997). Superoxide dismutases (SOD) and Alkaline phosphatase (AKP), acting as detoxification enzymes, protects herbivores from the threaten caused by reactive oxygen species (ROS) and phosphate groups, respectively, in host plants (Funk, 2001; Mittler, 2002; Gao et al., 2013), and the availability of food sources could be increased by detoxifying toxic secondary metabolite substances (Karban and Agrawal, 2002).

Plants synthesize secondary metabolite substances that can have various negative impacts on herbivore physiology (Chen, 2008; Zhu-Salzman et al., 2008). ROS serve in defense mechanisms, as messengers and triggers of DNA mutation, lipid peroxidation, protein denaturation and membrane destruction (Low and Merida, 1996; Breusegem et al., 2001; Asada, 2006). To scavenge ROS, plants have developed complex enzymatic mechanisms. Peroxidase (POD) can aid in the pathogen and pest defense and wound-healing of plants (Lagrimini, 1991). Catalase (CAT) decomposes hydrogen peroxide (H_2_O_2_), which causes cytotoxicity (Matés and Sánchez-Jiménez, 1999; Slaughter and O’ Brien, 2000; Mittler, 2002). Peroxidase (POD) catalyzes the O_2_-dependent oxidation of *o*-diphenols, and also acts as a highly reactive intermediary whose secondary reaction is believed to undertake the task for pathogen and pest defense (Thipyapong et al., 2004). Thus, the amount and activity of detoxification enzymes in plant reveals their reaction and resistance to insects, which in turn reflects the risk to insect herbivores.

Although a number of theories have been proposed for how the diversity of plants influences herbivores, there is a scarcity of experimental tests. In this study, we examined the survival rates and oviposition of *B. tabaci* females in contexts of polyculture and monoculture. To compare abilities in digestion and detoxification for *B. tabaci* in choice situations, which included plants from four species (polyculture) and a monoculture situation with four same plants (monoculture), we analyzed the concentrations of protein, trehalose, trehalase, SOD, AKP, sucrase and amylase. The activities of several detoxification enzymes in host plants from multi-plant treatment, from the single-plant treatments and from a control CK (plants without whiteflies) were measured to investigate the relationship between reaction of plant and insect feeding.

## Materials and Methods

### Plants and Insects

Seeds of the four species, tomato (*Solanum lycopersicum* Miller, var. ‘Saijinpeng’), summer squash (*Cucurbita pepo* L., var. ‘Xinzaoqing 1’), Chinese cabbage (*Brassica pekinensis* L., var. ‘Qinza 2’), and kidney bean (*Phaseolus vulgaris* L., var. ‘Didouwang’) were germinated at 26 ± 2°C for 3 days on moist cotton in 10 cm diameter petri dishes. Then, seedlings at the same stage of development were grown in 10 cm or 2 cm diameter plastic pots (one seedling per pot) with soil mixture (peat moss: perlite: vermiculite = 4: 1: 1). Temperature was 22 ± 2°C (night) to 27 ± 2°C (day) in a walk-in growth chamber (RXZ; Jiangnan Instrument Factory, Zhejiang, China; RH 60 ± 5%, light/dark 14:10 h, light intensity 1500–1800 Lux). Plants with four leaves were gently cleaned with water to prevent potential effect of impurities (soil on the leaves).

*Bemisia tabaci* adults were retrieved from their colony in cotton (*Gossypium hirsutum* L. var. ‘Shiyuan’) plants (Key Laboratory of Applied Entomology, Yangling, Shaanxi, China). Cotton, grown in 10 cm plastic pots at three-leaf stage, were placed in 40 × 40 × 40 cm cages with *B. tabaci* adults [Middle East-Asia Minor 1 (MEAM1), formerly B biotype, previously cultured on cotton for more than 7 generations] for 48 h for oviposition. Then, adults of whitefly were removed and the eggs were allowed to develop on cotton leaves. After 3 weeks, and for a 72 h window, newly emerged adults were collected and starved for 6 h before further work.

### *Bemisia tabaci* Female Adult Survival and Number of Eggs

There were five different treatments in total: a polyculture treatment and four monoculture treatments (tomato, Chinese cabbage, summer squash and kidney bean). The polyculture treatment consisted of each of the four plant species in a 40 × 40 × 40 cm cage with four potted plants, while four pots of the same plant species were in the monoculture treatments. Seedlings used were in 10 cm diameter plastic pots. Seventy-five couples of whitefly adults were put into each of the cages, 27 ± 2°C in day, 22 ± 2°C at night, RH 60 ± 5%, light/dark 14:10 h, light intensity 1500–1800 lux. All cages were shaken slightly and the position of plants randomly altered on a daily basis to ensure whiteflies move among different plant species. The blank control was as described above except that there were no whiteflies in cages. Number of female adults was counted every 24 h until day 7. 7 days later, number of eggs on leaves was counted under the stereomicroscope. All the experiments were replicated 5 times. Total number of eggs was the summation of eggs on all plants in one cage. In the polyculture treatment, number of eggs per pot of plants was the average number of eggs laid on the same kind of plant species in different cages. In the monoculture treatments, number of eggs per pot of plants was a quarter of the summation of number of eggs in the same cage.

### Analyses of Nutrition Enzyme, Detoxification Enzyme, Trehalose and Protein of *B. tabaci* Adult

Seven days after inoculating, the 40 surviving *B. tabaci* adults in single cages were ground in 1.5 mL tubes in liquid nitrogen, each to be considered a sample, and 400 uL of 0.1 M phosphate buffer solution (PBS) were added. The mixture was centrifuged at 1,500 × *g* for 20 min at 4°C and the supernatant was kept at -20°C before using for determining the content of sucrase, trehalase, amylase, SOD, AKP, trehalose, and total protein of *B. tabaci* adults. All experiments were replicated 5 times.

The dinitrosalicylic acid reagent (DNS) method was adopted to determine trehalase, amylase, and sucrase activities in whiteflies (Sumner, 1925). The activity of trehalase or sucrase was defined as μM glucose per min per mg protein at 37°C. Amylase activity was expressed as μg per mL maltose per min per mg protein. SOD activity was measured by WST-1 [2-(4-iodophenyl)-3-(4-nitrophenyl)-5-(2,4-disulfophenyl)-2H-tetrazolium, monosodium salt] method (Peskin and Winterbourn, 2000). And one unit of SOD activity was defined as the quantity of enzyme reached 50% inhibition in 1 mg total protein at 37°C. The analysis of AKP was referred to Funk (2001) and Zhang et al. (2014). The amount of trehalose was assayed with the anthrone method (Larsen et al., 1987). Total protein was measured using the Bradford method (Rodriguez-Saona et al., 2010). In the experiments, the microplate reader (M_200_, Tecan, Männedorf, Switzerland) and 96-well plates were used.

### Olfactometer Behavior Analysis

The Y-olfactometer was used in the olfactory response experiments of *B. tabaci*. The system consisted of an air pump, granular activated carbon, two gas flowmeters, two odor source bottles, and a Y glass tube (inside diameter 0.8 cm, arm length 10 cm, included angle 60°). All components were connected with silicone tubes. Seedlings grown in 2 cm diameter pots were used. There were four different treatments (tomato vs. mixture, Chinese cabbage vs. mixture, kidney bean vs. mixture, and summer squash vs. mixture). For each treatment, four pots of plants were put into each odor source bottle. One consisted of four pots of the same plant species (monoculture) while the other consisted of four pots of the different plant species (polyculture). The whiteflies were allowed to choose between two different odor sources. And the air was controlled as to pass through the system for 10 min prior to the test, with a flow rate of 50 mL/s (Honda et al., 1998).

One female whitefly adult was put into the middle of the Y-tube and was given 3 min for selection in each assay. The effective choice was identified if this whitefly entered more than 4 cm of one arm and remained for more than 30 s. Otherwise, it was recorded as an ineffective response. Each individual was used once only. The Y-olfactometer was placed on a white board under the fluorescent lamp (27 ± 2°C, 1 500 Lux). The position of the two arms was changed every four tests and the Y-tube was cleaned with distilled water every four tests. There were 70 effective tests for each treatment.

### Analyses of Enzyme Activity of Host Plants

To compare PPO, POD, CAT, and SOD in host plants from different treatments, 0.4 *g* fresh leaves with similar position of the plants, were detached (0.1 *g* from each pot of the four same species of plants in the monoculture treatments, 0.4 *g* from each pot of the four different plants in the polyculture treatment) as treated as one sample. Next they were separately ground into powder in porcelain mortars with liquid nitrogen. Then it was transferred to 10 mL centrifuge tubes containing 3.6 mL 0.05 M PBS centrifuged at 2,000 × *g* for 25 min at 4°C, and transferred to 1.5 mL tubes and held at -20°C before use.

For PPO assays, the catechol method was used, and PPO activity was determined as ΔOD_410_ per min per g fresh weight (Ma et al., 2008). For POD, methyl catechol and hydrogen peroxide were used. The activity of POD was expressed as ΔOD_470_ per min per *g* fresh weight (Deng et al., 2013). Slaughter method was used for analyzing the activity of CAT (Slaughter and O’ Brien, 2000). OD_240_ of a mixture of 20 μL supernatant and 980 μL 6.25 mM H_2_O_2_ was measured every 15 s in an ultraviolet spectrophotometer (NanoDrop 2000, Thermo, Boston, Massachusetts, United States), 11 times. ΔOD_240_ per min per g fresh weight was used to define the activity of CAT (Zhang et al., 2014).

### Data Analyses

Data analyses was conducted using SPSS (17.0, IBM, Chicago, IL, United States). Specifically, the data on *B. tabaci* adult survival, total number of eggs, and biochemical test were analyzed with ANOVA, and the average were analyzed using the least significant difference test (LSD) when the homogeneity of variance was met. Independent *t*-tests were used to distinguish the differences between number of eggs per pot in the polyculture treatment and the monoculture treatment. Number of eggs in the polyculture treatment were analyzed with the Friedman-Test. Pearson bivariate correlation analysis was used to test for any correspondences between indices. Data of Y-olfactometer behavior response was analyzed with χ^2^-test.

## Results

### *Bemisia tabaci* Female Adult Survival and Number of Eggs

**FIGURE 1:**
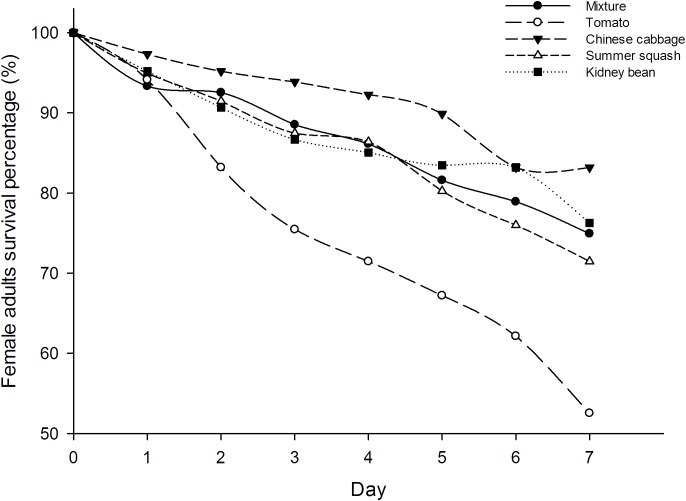
Survival (%) of *Bemisia tabaci* female adults in the polyculture treatment and four monoculture treatments.

The survival rates of *B. tabaci* female adults in five different treatments were not significantly different at day one (*F* = 1.16; *df* = 4, 20; *P* = 0.36), but were so at all subsequent days up to 1 week (*F* = 6.25, 14.11, 17.51, 9.87, 19.11, 22.28; *df* = 4, 20; *P* = 0.002, 0.000, 0.000, 0.000, 0.000, 0.000). The survival rate of whiteflies in tomato treatment were the lowest from the second day. Seven days after inoculation, the survival rate of *B. tabaci* in the polyculture treatment was 74.9%, which was higher than those in the tomato treatment (52.5%, *P* = 0.000) while lower than those in the Chinese cabbage treatment (83.2%, *P* = 0.027). However, there were no significant differences between the survival of female adults in polyculture and summer squash treatment (71.5%, *P* = 0.327), or kidney bean (76.3%, *P* = 0.703). The survival rate of whiteflies in polyculture was roughly an average of all treatments. In the four monocultures, the highest survival rate was observed in Chinese cabbage, and the lowest was in tomato (**Figure 1**).

**FIGURE 2:**
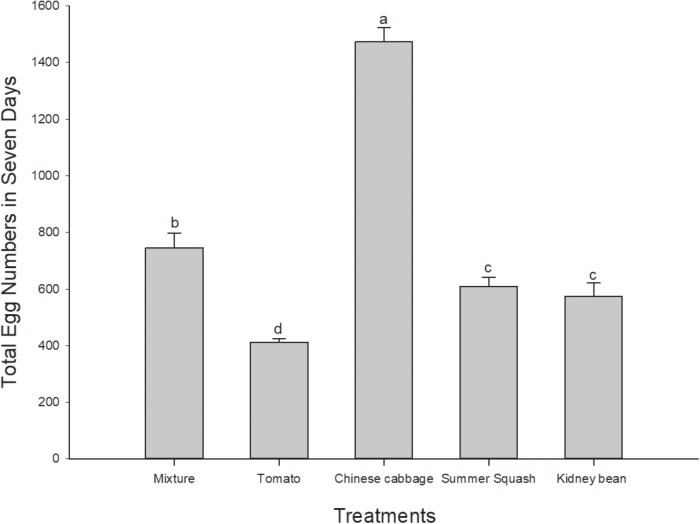
Total numbers of eggs of *B. tabaci* fed for 7 days in the polyculture treatment and four monoculture treatments. Rows with different lowercase letters differ significantly at *P* < 0.05 (ANOVA-LSD).

In this 7 day period, total number of eggs of *B. tabaci* female adults in the Chinese cabbage treatment was the highest of the five treatments (*F* = 98.43; *df* = 4, 20; *P* = 0.000). The number of eggs in polyculture treatment was higher than that of summer squash (*P* = 0.032) and kidney bean (*P* = 0.009), while the number in tomato treatment was significantly lower than that of summer squash (*P* = 0.003) and kidney bean (*P* = 0.013) (**Figure 2**).

**FIGURE 3:**
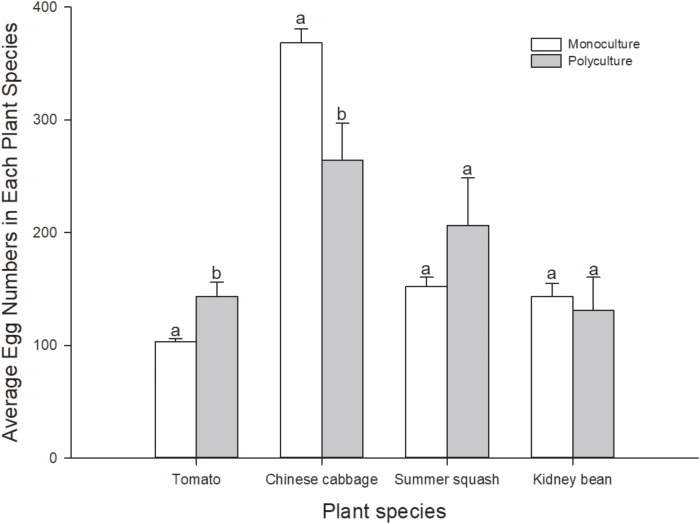
Number of eggs per pot of plants of *B. tabaci* fed for 7 days in the polyculture treatment and four monoculture treatments. Paired means ( ± SE; *n* = 5) with the same lowercase letters are not significantly different (Independent *t*-test: *P* > 0.05).

Number of eggs per pot of plant in polyculture and monoculture treatments was also calculated. In polyculture, *B. tabaci* laid more eggs on individual plants of Chinese cabbage and summer squash than on tomato and kidney bean (Friedman χ^2^ = 7.80, *df* = 3, *N* = 5, *P* = 0.050). In monoculture treatments, more eggs were laid on Chinese cabbage, while number of eggs on kidney bean and summer squash were lower than Chinese cabbage, and those on tomato were the lowest (*F* = 155.3; *df* = 3, 16; *P* = 0.000). Number of eggs per pot of the same plant species in the polyculture treatment and monoculture treatments were compared. Number of eggs per plant in polyculture was significantly higher than that in tomato treatment (*t*_8_ = 3.130; *P* = 0.030), and significantly lower than that in Chinese cabbage treatment (*t*_8_ = -2.957; *P* = 0.018), but not significantly different in summer squash and kidney bean treatments (*t*_8_ = 1.264, -0.391; *P* = 0.242, 0.711) (**Figure 3**). Correlation analysis indicated that number of eggs per plant on four different plant species in polyculture treatment had no correlation with that in monoculture treatments (*r* = 0.880; *N* = 4; *P* = 0.120).

### Biochemical Analysis of *B. tabaci* Adults

The levels of total protein in whiteflies in the polyculture treatment were not significantly different from those in tomato, summer squash, and kidney bean. While the level in Chinese cabbage was significantly higher than that in polyculture (*P* = 0.000). The concentration of trehalose and SOD in whitefly from the polyculture treatment was significantly higher than Chinese cabbage (*P* = 0.021, 0.027), and significantly lower than those from the tomato treatment (*P* = 0.033, 0.038), while they were not significantly different from those in the summer squash and kidney bean treatments. Moreover, the activity of AKP in whitefly from the polyculture showed no significant difference from monoculture in the case of tomato, summer squash and kidney bean, but was significantly lower than that in the Chinese cabbage treatment (*P* = 0.000).

The activity of trehalase in whitefly adults from the polyculture treatment was significantly lower than those from tomato and kidney bean treatments (*P* = 0.020, 0.002) at the 7th day, but similar to those from Chinese cabbage and summer squash. Furthermore, the sucrase in *B. tabaci* from the polyculture treatment was similar to those from tomato, Chinese cabbage, and summer squash, but was significantly higher than in kidney bean (*P* = 0.011). The activity of amylase in whiteflies from polyculture treatment was similar to those from monoculture treatments, except for Chinese cabbage (*P* = 0.001) (**Table 1**).

**Table 1:** Concentration of total protein, trehalose, superoxide dismutases (SOD) and alkaline phosphatase (AKP), and activity of trehalase, sucrase, and amylase of *Bemisia tabaci* adults fed for 7 days in five different treatments.

	Mixture	Tomato	Chinese cabbage	Summer squash	Kidney bean	*F*_4,20_	*P*
Protein mg/mL	125.1 ± 8.274bc	111.6 ± 2.945c	172.1 ± 9.557a	125.7 ± 5.269bc	133.4 ± 5.175b	11.74277	<0.001
Trehalose mmol/L	93.67 ± 15.62b	133 ± 15.33a	50.74 ± 7.709c	95.47 ± 10.79ab	100.4 ± 9.091ab	5.82488	<0.05
SOD U/mg protein	69.52 ± 4.578b	80.45 ± 2.54a	57.78 ± 3.38c	73.46 ± 3.26ab	64.72 ± 3.351bc	6.083047	<0.05
AKP U/g protein	2.645 ± 0.2849bc	3.119 ± 0.2736b	4.843 ± 0.297a	2.273 ± 0.2605bc	1.816 ± 0.2795c	17.45213	<0.001
Trehalase mmol/L/mg protein/min	3.446 ± 0.203c	4.137 ± 0.1386ab	3.505 ± 0.2715c	3.602 ± 0.1663bc	4.408 ± 0.1619a	4.879462	<0.05
Sucrase mmol/L/mg protein/min	24.02 ± 2.187a	24.82 ± 2.625a	27.24 ± 1.492a	24.75 ± 1.976a	16.3 ± 1.148b	4.532243	<0.05
Amylase mg/mL/mg protein/min	25.19 ± 1.977a	24.76 ± 1.783a	16.6 ± 0.7479b	23.93 ± 1.707a	23.4 ± 0.6927a	5.61991	<0.05


*Rows with different lowercase letters differ significantly at *P* < 0.05 (one-way ANOVA).*

Among the five different treatments, survival rate of *B. tabaci* had significant negative correlation with its trehalose and SOD activity (*r* = -0.902, -0.928; *N* = 5; *P* = 0.037, 0.023). The trehalose and amylase in *B. tabaci* adults had significantly negative correlation with their oviposition (*r* = -0.944, -0.928; *N* = 5; *P* = 0.016, 0.023). While number of eggs positively correlated with the total protein in whiteflies (*r* = 0.960; *N* = 5; *P* = 0.010) and trehalose had significantly positive correlation with SOD (*r* = 0.904; *N* = 5; *P* = 0.035).

### Y-Shape Olfactometer Behavior Analysis

**FIGURE 4:**
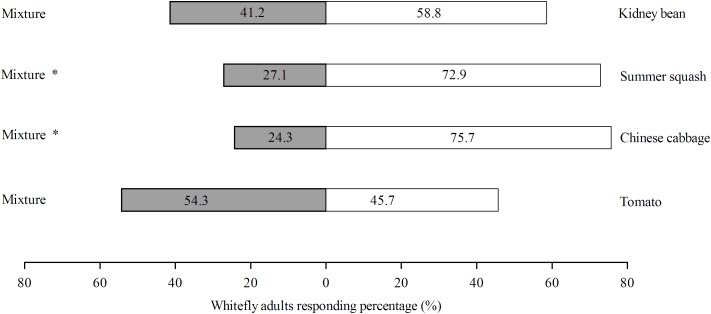
Olfactory responses (%) of *B. tabaci* female adults toward polyculture treatment vs. monoculture treatment within 3 min. There were four pots of plants in each odor source bottle. One consisted of four pots of same plant species (monoculture) while the other one is a mixture of four pots of four different plant species (polyculture). Behavior response was analyzed with χ^2^-test, with ‘^∗^’ denoting significant difference (*P* < 0.05). ‘% response’ indicates the percentage of whiteflies responding to odor sources within 3 min.

Of the four different treatments, the response rates of *B. tabaci* were 75.3, 68.6, 65.4, and 82.4% for Chinese cabbage vs. mixture, tomato vs. mixture, and kidney bean vs. mixture, respectively. For the choice experiments, *B. tabaci* female adults preferred polyculture over monoculture in the case of Chinese cabbage (*χ^2^* = 18.51; *df* = 1; *P* = 0.000) and summer squash *(χ^2^* = 14.63; *df* = 1; *P* = 0.000), but did not differentiate polyculture and monoculture in the case of tomato (*χ^2^* = 0.514; *df* = 1; *P* = 0.473) and kidney bean (*χ^2^* = 2.057; *df* = 1; *P* = 0.151) (**Figure 4**).

### Activities of Detoxification Enzymes in Plants

**FIGURE 5:**
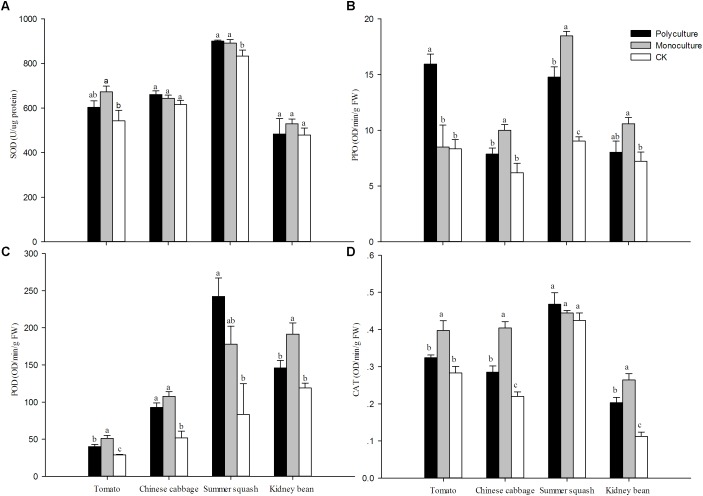
Relative activities of **(A)** superoxide dismutases (SOD), **(B)** polyphenol oxidase (PPO), **(C)** peroxidase (POD) and **(D)** catalase (CAT) in different host plants. Means (± SE; *n* = 5) with the same lowercase letter are not significantly different (ANOVA-LSD: *P* > 0.05). Polyculture: Four pots per cage with one of each plant species and 75 couples of *B. tabaci* adults were released; Monoculture: Four pots per cage with the same plant species and 75 couples of *B. tabaci* adults were released; CK: Undamaged plants.

The activities of SOD, PPO, POD, and CAT of host plants with *B. tabaci* were higher relative to plants without whiteflies in nearly all cases. However, differences in activity were not observed for CAT in summer squash, SOD in Chinese cabbage and kidney bean. For CAT in Chinese cabbage and kidney bean, POD in tomato, and PPO in summer squash, the trend observed was monoculture>polyculture>CK. For POD in kidney bean, CAT in tomato, and PPO in Chinese cabbage, the trend was monoculture>polyculture=CK. For SOD in summer squash and POD in Chinese cabbage, it was monoculture=polyculture>CK. For SOD and CAT, the activities were high in polyculture and CK, and in monoculture for summer squash (**Figure 5**). In the four monoculture treatments, correlation analysis showed that the activity of sucrase in *B. tabaci* adults had a positive correlation with CAT, for different host plants (*r* = 0.910; *N* = 4; *P* = 0.089).

## Discussion

In grassland and other non-forested systems, plant diversity has been manipulated in order to increase biomass (Cardinale et al., 2011), but diversity changes have cascading consequences for higher trophic levels (Cook-Patton et al., 2011, 2014). Polyphagous herbivores, faced with an often complex and changing environment, have the option of a variety of plants and nutrient types, choices critical to competitive ability and survival. *B. tabaci* faces fierce competition with native insects in the search for space and host plants. Like other herbivores, choices made by *B. tabaci* impact their survival (Zhang et al., 2014).

This experiment indicated that the longevity and oviposition of female adults of *B. tabaci* in the multi-plant treatment were an average of the five treatments. In 4 day preliminary experiments, conducted in petri dishes with four excised leaves (one leaf each of the different plant species for polyculture, or all of the same plant for monoculture), we also found that the survival rate of *B. tabaci* adults was an average in comparison to the monocultures. In the experiments reported herein, whitefly oviposition was greatest on Chinese cabbage both in polyculture and monoculture. Comparing tomato and kidney bean, more eggs were laid on the former in the case of polyculture, although in monoculture tomato had the fewest. Cucumber and tomato were preferred for oviposition by *B. tabaci* compared to three other species of plants (Zhang et al., 2014). However, in this assay, Chinese cabbage was most favored by female adults, which showed the highest survivorship and oviposition among treatments both for polyculture and monoculture. This was probably because cucumber is an optimal host for foraging and oviposition of *B. tabaci* (Shah and Liu, 2013). Based on the foraging and oviposition hypotheses, the plant species optimal for feeding or reproduction might not be the same (Costa et al., 1991; Zhang et al., 2014). Moreover, the locomotion ability of *B. tabaci* females was higher in the multi-plant treatments, where they might spend more time and consume more energy on host plant selection (Bernays, 1999), while at the same time, there might be a greater chance that they would find favorable hosts for oviposition compared to monoculture (Zhang et al., 2014).

Concentrations of total protein and trehalose in *B. tabaci* were not significantly different between the multi-plant treatment and the tomato, summer squash and kidney bean monocultures, although the total protein in Chinese cabbage monoculture was higher. Importantly, analysis of detoxification enzymes showed that the activity of AKP in *B. tabaci* from the multi-plant species treatment, for which the second highest oviposition rate was observed, was significantly lower than that of tomato and Chinese cabbage single-plant treatments, while more eggs were oviposited on Chinese cabbage. The same trend was observed with SOD activity, in that *B. tabaci* adults from multi-plant treatments was lower than in tomato. Lower concentrations of AKP and SOD would translate to a reduced rate in phosphate monoester and ROS catalyzation, which would mean the host plant would be less toxic for whitefly adults in polyculture and Chinese cabbage monoculture. Moreover, in the five different treatments, survival rate was negatively correlated with trehalose and SOD activity. This likely explains why Chinese cabbage monoculture showed larger survival rate and egg numbers. When *B. tabaci* performed poorly in fecundity, the concentration of trehalose in whiteflies was higher, indicating that the Chinese cabbage monoculture and the polyculture were the host preferred by the insect.

Since the amount of trehalose and the activity of amylase were negatively correlated with oviposition by *B. tabaci*, and the oviposition rate in polyculture was relatively high, female adults in polyculture appear somehow distracted in multi-host situations, with a greater amount of time spent on host selection. These results reflect Chu et al. (2012), who suggested that population fitness of *B. tabaci* is adversely impacted by the presence of a non-preferred host. In our assay, tomato was the least attractive to whiteflies. Detoxification enzymes were in greater concentration in *B. tabaci* from tomato than Chinese cabbage monoculture, and the polyculture treatment, which was probably due to high PPO activity in tomato. In this study, oviposition per plant on tomato, kidney bean and summer squash in the polyculture treatment were lower than those in monoculture. This might be observed because *B. tabaci* were highly mobile and spent longer on their prefered hosts (such as Chinese cabbage), thus with greater feeding and oviposition on those. This would seem to be in support of both the optimal foraging theory and the optimal oviposition theory.

The processes underlying the positive effect that the richness of plant species has on herbivore abundance may be related to variation or increases in plant resources and vegetational structure (Haddad et al., 2001). A certain combination of nutrients from varied host plants would probably enhance the adaptability of *B. tabaci* (Funk, 2001; Mittler, 2002; Zhang et al., 2008, 2014). Increased expression of digestive enzymes in adults of *B. tabaci* enables greater access to nutrition (Jermy, 1984), although the concurrent increase in exposure to plant secondary metabolites means that there is a trade off for energy balance. This is reflected in the current findings on survival rate and oviposition in the polyculture compared with Chinese cabbage monoculture. Moreover, the greater plant diversity of natural systems means much greater abundance of natural enemies, more physical restrictions of movement, and masking from non-host plants, all of which can decrease pest populations (Lal, 2016).

Moreover, the ‘enemy hypothesis’ suggests that polyculture would attract more insect predators and would be more efficient in herbivore control (Root, 1973). Polyculture decreases predator emigration and local extinctions, and might provide greater supplies of pollen and nectar resources (Root, 1973). For instance, populations of aphids and thrips were significantly lower when the plants cowpea and sorghum were intercropped, although with higher abundances in the case of cowpea and greengram polycultures (Nampala et al., 2002). Intercropping is a common method of disrupting activity of insect pests and attracting their natural enemies. For example, when *Vicia faba* L. was intercropped with *Ocimum basilicum* L. or *Satureja hortensis* L., the population of *Aphis fabae* Scop. was significantly lower than monoculture (Basedow et al., 2006). Similar strategies could be applied in greenhouses to manage whiteflies, for instance, eggplants can attract *Trialeurodes vaporariorum* Westwood from *Euphorbia pulcherrima* Willd. ex Koltz (Lee et al., 2010). The attractiveness of tomato volatile substances for *B. tabaci* can be interfered by *Coriandrum sativum* L. volatiles, as a result the abundance of whiteflies on tomatoes is reduced (Togni et al., 2010). These results are consistent with the results of the olfactometer behavior analysis herein. And with the development of modern farming strategies, there is growing concerns about the sustainability of intensified agricultural systems, and intercropping is gaining attention as an approach to alleviate resulting increases in insects pest damage. Compared with monoculture, polyculture has higher plant species richness, a lower reliance on disturbance, and a smaller reliance on exogenous sources of nutrition (Crews, 2005; Young, 2015).

Generally, the multi-plant diet was more beneficial to *B. tabaci* comparing to the monoculture of tomato and summer squash, although Chinese cabbage monoculture was the preferred diet. This result can be translated in terms of cropping strategies in greenhouses for management of *B. tabaci*. On the one hand, when whiteflies are reared as prey for natural enemies, such as *Orius sauteri* and *Encarsia formosa*, using Chinese cabbage or polyculture would provide higher prey densities for the culturing of natural enemies. On the other hand, for certain crops, intercropping will likely be a successful strategy for interrupting whiteflies pests. As suggested by Bird and Krüger (2006), *B. tabaci* appears to have trouble in host choice when lower ranking hosts were present. Moreover, in this work, we used separately planted hosts, as to eliminated interference in below ground plant interactions. In future work, *B.tabaci* mobility, plant inter-species interactions and heterogenity of resources (Moreira et al., 2016) should also be taken into consideration.

## Author Contributions

ND, KZ, and T-XL designed the assay. ND and KZ conducted the experiments and wrote the manuscript. SW, FZ, and KZ analyzed the data. All authors revised the manuscript.

## Conflict of Interest Statement

The authors declare that the research was conducted in the absence of any commercial or financial relationships that could be construed as a potential conflict of interest.
